# Migration Routes and Strategies in a Highly Aerial Migrant, the Common Swift *Apus apus*, Revealed by Light-Level Geolocators

**DOI:** 10.1371/journal.pone.0041195

**Published:** 2012-07-18

**Authors:** Susanne Åkesson, Raymond Klaassen, Jan Holmgren, James W. Fox, Anders Hedenström

**Affiliations:** 1 Department of Biology, Lund University, Lund, Sweden; 2 British Antarctic Survey, Natural Environment Research Council, Cambridge, United Kingdom; University of Durham, United Kingdom

## Abstract

The tracking of small avian migrants has only recently become possible by the use of small light-level geolocators, allowing the reconstruction of whole migration routes, as well as timing and speed of migration and identification of wintering areas. Such information is crucial for evaluating theories about migration strategies and pinpointing critical areas for migrants of potential conservation value. Here we report data about migration in the common swift, a highly aerial and long-distance migrating species for which only limited information based on ringing recoveries about migration routes and wintering areas is available. Six individuals were successfully tracked throughout a complete migration cycle from Sweden to Africa and back. The autumn migration followed a similar route in all individuals, with an initial southward movement through Europe followed by a more southwest-bound course through Western Sahara to Sub-Saharan stopovers, before a south-eastward approach to the final wintering areas in the Congo basin. After approximately six months at wintering sites, which shifted in three of the individuals, spring migration commenced in late April towards a restricted stopover area in West Africa in all but one individual that migrated directly towards north from the wintering area. The first part of spring migration involved a crossing of the Gulf of Guinea in those individuals that visited West Africa. Spring migration was generally wind assisted within Africa, while through Europe variable or head winds were encountered. The average detour at about 50% could be explained by the existence of key feeding sites and wind patterns. The common swift adopts a mixed fly-and-forage strategy, facilitated by its favourable aerodynamic design allowing for efficient use of fuel. This strategy allowed swifts to reach average migration speeds well above 300 km/day in spring, which is higher than possible for similar sized passerines. This study demonstrates that new technology may drastically change our views about migration routes and strategies in small birds, as well as showing the unexpected use of very limited geographical areas during migration that may have important consequences for conservation strategies for migrants.

## Introduction

Long-distance migration by birds is typically carried out as cycles of fuelling at stopovers followed by flight towards the next suitable stopover [1]. In some extreme cases the whole migration is covered in one flight step, such as in the Alaskan bar-tailed godwits *Limosa lapponica baueri* [2], [3]. This strategy has probably evolved as a response to non-uniform distributions of food limited to specific habitats, combined with the need to cross wide ecological barriers like seas and deserts. This migratory strategy involves not only the deposition of large fuel reserves but also associated physiological changes such as temporarily enlargement and shrinkage of flight muscles and organs involved in food assimilation [4]. During periods of extensive fuelling, predation risk may increase as a result of reduced manoeuvrability due to heavy fuel loads [5]–[7]. An alternative migration strategy involves short flights with small fuel reserves, which avoids the costs of carrying heavy fuel loads but instead requires many stopovers and availability of suitable habitats along the migration route. Other birds, like seabirds, raptors and terrestrial species feeding on aerial insects, may instead use a fly-and-forage migration strategy [8], without the need of extensive stopover periods if their food is more evenly distributed and available along the migration route. Note that a fly-and-forage strategy may be combined with stopovers as observed in certain seabirds [9], [10], so that not all energy required for migration is acquired during migratory movement itself. How migrants organize their travels in relation to environmental conditions can only be resolved if we are able to track individual birds throughout their migration, but until recently this has been limited to relatively large birds that can sustain the weight of a satellite transmitter or GPS logger. The tracking of small (avian) migrants has only recently become possible by the use of retrievable archival geolocator units (e.g. [10], [11]), which record time and light-level data allowing for the reconstruction of time-stamped latitudes and longitudes. In the present study we successfully recorded the full migration of common swifts *Apus apus* (henceforth called swift) from two breeding sites in Sweden. The swift is a highly aerial species, only leaving its aerial habitat during the breeding season and during occasional roost events in trees [12]. Non-breeding birds roost on the wing during the night [13], and during migration and wintering they are believed to spend all their time airborne [14]–[16]. The migration routes used by swifts and their wintering areas in tropical Africa are to a large extent unknown since only a few ringing recoveries have been reported from south of the Sahara thus far [17]–[19]. Here we describe the details of the migrations of individual swifts, discuss the migration strategy for this enigmatic aerial migrant, and compare with other migrants following the typical migration strategy of terrestrial birds.

## Results

### Migration Routes and Wintering Area

**Figure 1:**
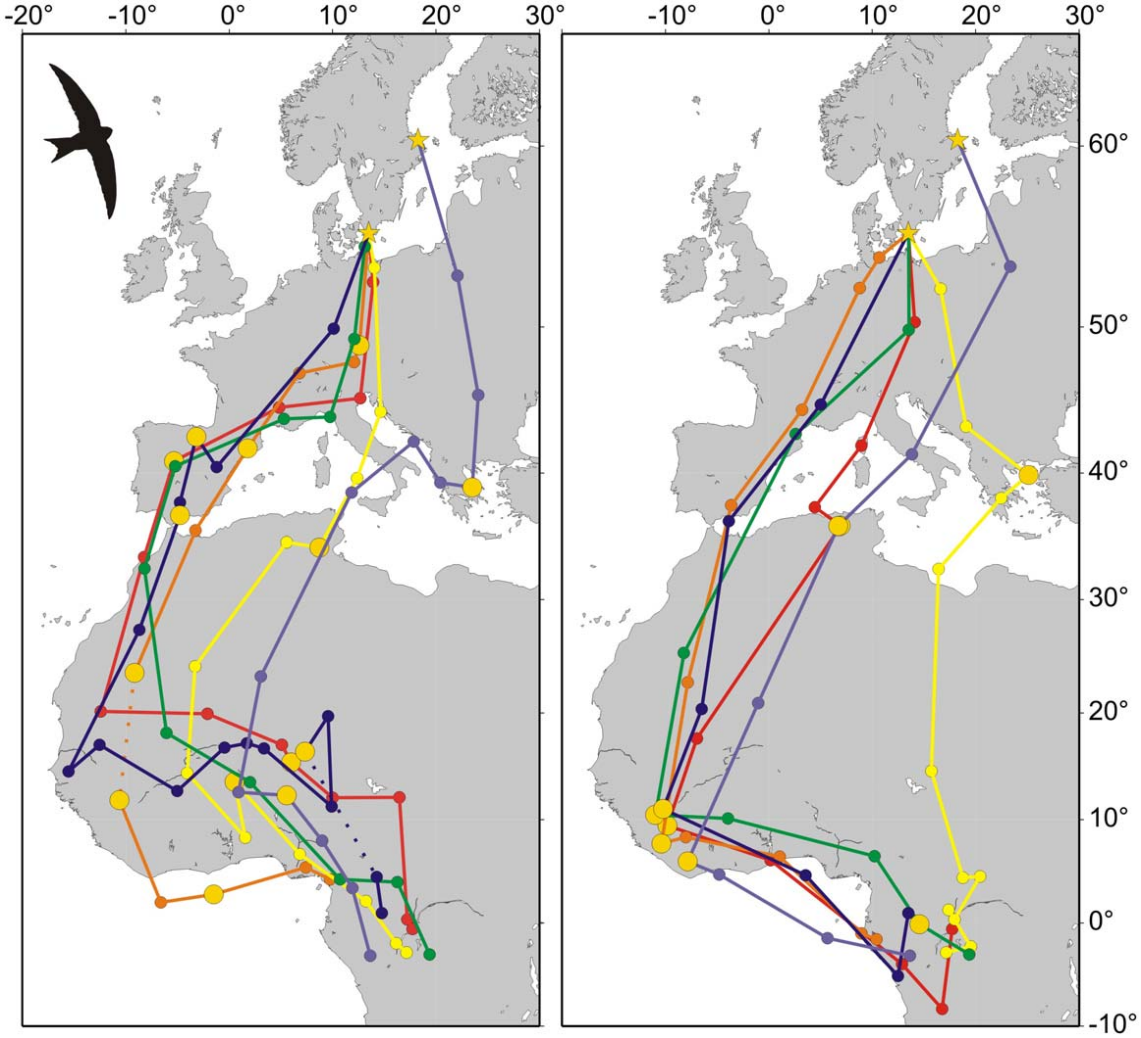
Migration tracks of swifts. (A) Autumn migration tracks for 6 individuals where filled circles represent 3-day average positions and filled yellow circles represent stopover periods when the bird did not move (2 days or more). Dotted lines indicate lack of data around autumn equinox. (B) Spring migration tracks for the same birds as in (A).

The autumn mean initial migration direction was 182° for the tracked birds, with one bird taking a more easterly initial direction (Figure 1A). Four of the birds shifted to a direction towards SW through Europe to reach Africa via Gibraltar, while two crossed the Mediterranean via the Balkan and/or Apennine Peninsulas to arrive in Africa near Cap Bon, Tunisia (Figure 1A). Within West Africa migration proceeded towards south, while the two individuals entering Africa near Cap Bon proceeded towards SSW. Five of the birds aggregated in Central West Africa (latitudes 5.97°N–11.05°N; longitudes 7.85°W–11.99°W), with one bird taking a more southerly route towards the final wintering area in Central Africa that involved an open sea crossing (Figure 1A). Five of the birds made stopovers in West Africa, lasting between 10 and 56 days, before proceeding to the wintering area in Central Africa.

**Figure 2:**
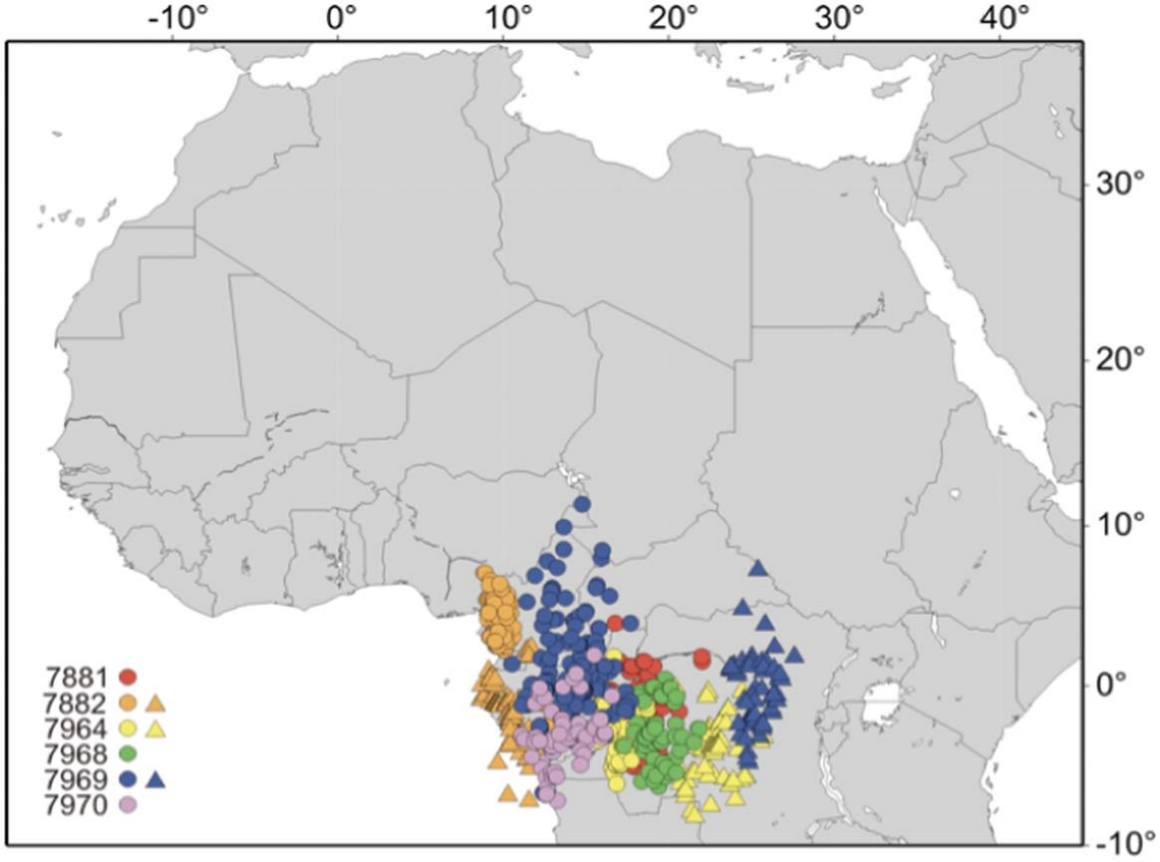
Winter locations for swifts. Symbols represent daily positions of 6 individual swifts during the winter period, with colours showing individual locations. Three individuals (7882, 7964 and 7969) changed location during the winter as indicated by triangles. Details for each individual are found in Figure S1.

All six birds spent the winter in the same general area in Central Africa between latitudes 0.97°N–3.20°S and longitudes 10.42°E–19.38°E (Figure 2), during a period of on average 198 days (range 172–243 days). Three of the birds shifted location during the winter (Figure 2), while one of these (7969) returned to the area where it had spent the first part of the winter.

Spring migration routes were similar to the autumn routes, while five of the birds visited a rather restricted area in SW West Africa (Liberia), where they stopped over for on average 7 days (SD = 4.1, range 2.5–11.5 days), before continuing towards NNE across Sahara (Fig. 1B). One bird, breeding in the colony in southern Sweden (see Methods), took a more direct route through Central Sahara and crossed the Central Mediterranean to reach the Balkan Peninsula on the way north (Figure 1B). The overall migration direction through Europe was NNE in five of the birds, except the individual arriving in Balkan, from where it took a NNW-direction towards its breeding site in southern Sweden. The individual breeding at the northern site shifted from NNE towards NNW during the last migration leg that involved a flight across the Baltic Sea (Figure 1B).

The autumn migration route was on average 53% longer than the direct route between the breeding site and the wintering area, while it was slightly more direct during spring migration with a 43% detour. The difference between autumn and spring detours was however not significant (Matched pair test, P>0.36).

### Migration Strategy

The duration of the entire autumn migration was on average 69 days (range 30–99 days), divided into 30 days of travelling and 39 days of stopover (i.e. stops >2 days, see Methods). The large variation was due to that three individuals spent lengthy stopovers of 56–82 days in West Africa (Table 1, Figure 1A). Corresponding numbers for spring migration was 29 days duration (range 18–34 days), divided between 21 days of travelling and 8 days at stopovers (Table 1). The number of travelling days did not differ significantly between autumn and spring (Matched pair, t = 1.82, P = 0.064), while number of days at stopovers did (t = 2.30, P = 0.035). Also the total duration of migration differed between the seasons with an average duration of 69 and 29 days (t = 3.46, P = 0.009), respectively. However, this difference is largely due to very long stopovers in three individuals during the autumn migration. Stopover periods were more evenly distributed along the migration route during autumn migration compared with the spring, when the stopovers were concentrated to sites in West Africa (Liberia) and after the Sahara crossing (Figure 1). Those individuals stopping for the shortest periods south of the Sahara before the northward spring migration (0, 2.5 and 2.5 days, respectively) were those that also stopped over in North Africa and the Balkan, while the birds stopping for longer periods south of the Sahara (7, 9.5, and 11.5 days) did not stop after having crossed the Sahara (P = 0.012, Mann-Whitney U-test).

**Table 1:** Average key numbers of migration for swifts *Apus apus* as recorded using light-level geolocators, N = 6.

Autumn migration	Average	Range
Departure from breeding area	2 August	28 July–12 August
Travel time (days)	30	18–47
Stopover time (days)	39	0–82
Total duration (days)	69	30–99
Migration distance (km)	9769	8629–12380
Direct distance (km)	6439	6061–6937
Detour (%)	53	33–104
Travel speed (km/day)	344	263–481
Migration speed (km/day)	170	87–302
**Wintering period**		
Arrival at wintering area	10 October	27 August–19 November
Departure from wintering area	26 April	23 April – 30 April
Duration of wintering period (days)	198	162–243
**Spring migration**		
Arrival at breeding area	25 May	12 May – 2 June
Travel time (days)	21	14 – 29
Stopover time (days)	8	4 – 13
Total duration (days)	29	18 – 34
Migration distance (km)	9208	7946–10390
Direct distance (km)	6439	6061–6937
Detour (%)	43	22–66
Travel speed (km/day)	469	274–650
Migration speed (km/day)	336	234–523

### Speed of Migration

Overall migration speed is the rate of travel when including time for actual movement and time for refuelling at stopovers [19]. An appropriate estimate of migration speed should therefore also include fuelling time at the breeding and wintering sites, but such information is impossible to achieve on the basis of tracking data. However, during a long inter-continental migration as in the swift the relative importance of the first fuelling episode at the breeding or wintering sites, if it exists, is relatively unimportant. Furthermore, fuelling loads seem rather small in the swift; five swifts captured immediately before onset of autumn migration weighed only 0.7 grams more than when captured soon after arrival at the breeding colony (see Methods).

**Figure 3:**
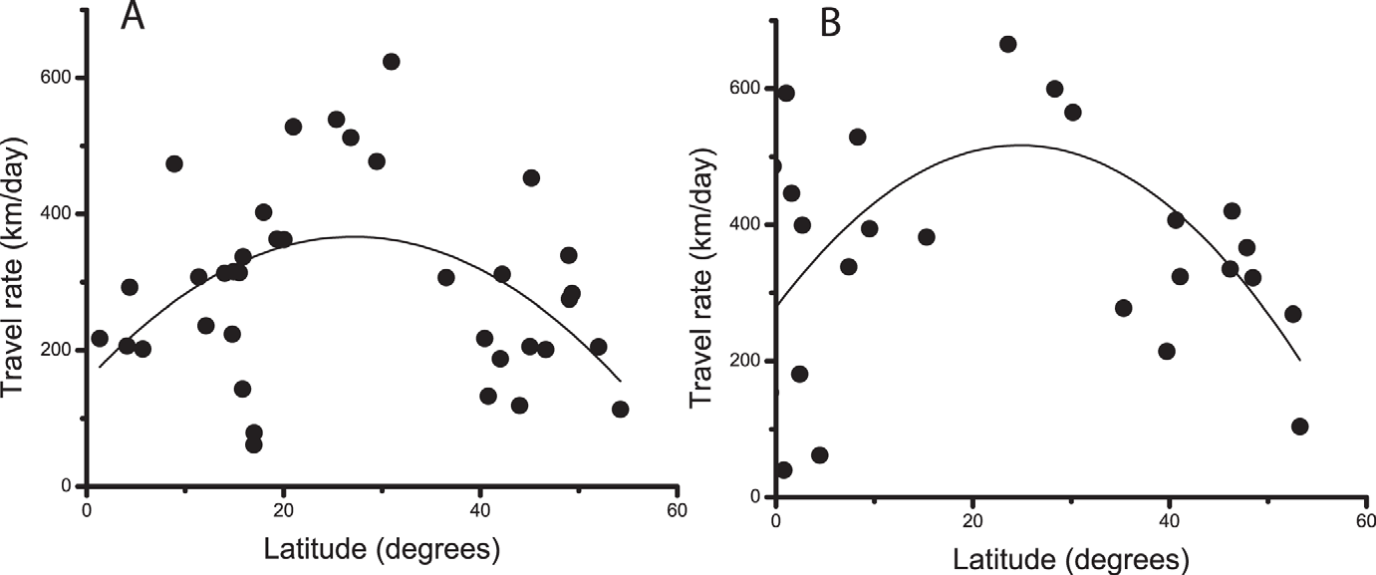
Travel rate for migrating swifts in relation to latitude. Daily travel rate in relation to latitude calculated for 3-day segments for six migrating swifts for periods of actual travel during (A) autumn and (B) spring migration, respectively. The curves show second degree polynomial fitted to the data: (A) U_trav_ = 155.5+15.6 Lat –0.29 Lat^2^, with maximum travel rate at latitude 27.1°N; (B) U_trav_ = 278.4+19.3 Lat –0.39 Lat^2^, with maximum travel rate at latitude 24.8°N.

The overall migration speed was significantly higher in spring (on average 336 km/day, Table 1) than in autumn (170 km/day, Table 1) (Matched pair test, t = 3.09, df = 5, P = 0.027). By excluding the stopovers we can calculate the rate of travel for the periods of movement (travel rate), which again was higher in spring (469 km/day, Table 1) than in autumn (344 km/day, Table 1), but the difference was not significant (t = 1.71, df = 5, P = 0.074). The travel rate (based on three-day averages) appears to show a non-linear relationship in relation to latitude in both seasons (Figure 3). A statistical model including latitude, latitude squared and season with individual as random factor showed that both latitude squared and season had significant effects on travel rate (Fixed effects; latitude*latitude: F_1,58_ = 20.3, P<0.0001; season: F_1,48_ = 9.5, P = 0.0034).

### Wind Assistance in Spring

The geolocators do not provide information about the altitude of the bird, thus we calculated wind assistance for different flight altitudes along the individual migration tracks for spring migration (Table 2). The five birds migrating via Liberia in spring experienced favourable wind assistance at most altitudes at the first migration leg between the wintering area and Liberia (Table 2), with particularly strong tail winds at high altitudes (3000 – 5000 m a.s.l.). The one bird that took a more direct route across the Sahara towards N, not travelling via Liberia, experienced head winds (negative wind assistance) at all altitudes during the Sahara crossing, while the birds migrating via Liberia could find favourable winds across the Sahara at most altitudes (Table 2). The bird that migrated directly towards the N from the wintering area in central Africa had the slowest migration speed (234 km/day) and speed of travel (274 km/day) among all the birds, although it had the shortest detour (Table S1). For the final migration leg across Europe there were mostly headwinds, sometimes at all altitudes, but two individuals experienced potential wind assistance (Table 2).

**Table 2:** Wind assistance for six swifts *Apus apus* during spring migration calculated for four different pressure levels (925, 850, 700 and 500 hPa, respectively) representing altitudes 750 m, 1500 m, 3000 m and 5000 m, respectively.

	Part 1, Congo-Liberia detour				Part 2, Crossing Sahara Desert				Part 3, Crossing Europe			
**Swift**	925	850	700	500	925	850	700	500	925	850	700	500
7881	0.6	3.0	6.6	6.1	1.1	2.1	4.8	8.3	**−4.4**	**−4.8**	**−5.2**	**−7.3**
7882	0.5	1.8	7.2	6.3	**−1.0**	0.9	5.8	9.3	**−0.3**	0.9	3.6	5.3
7964*					**−1.0**	**−0.8**	**−2.4**	**−4.6**	**−2.8**	**−4.1**	**−6.2**	**−8.4**
7968	**−0.2**	1.8	9.9	6.6	**−1.7**	1.8	5.9	8.9	**−0.2**	0.1	**−1.0**	**−6.1**
7969	1.5	3.5	6.6	7.4	**−0.5**	**−1.3**	**−2.7**	3.4	**−0.9**	0.0	**−0.3**	**−2.8**
7970	0.5	1.8	8.0	0.6	**−1.3**	0.5	3.0	4.1	4.7	3.7	3.1	6.7
**Mean**	0.6	2.4	7.7	5.4	**−0.7**	0.6	2.4	4.9	**−0.6**	**−0.7**	**−1.0**	**−2.1**

Tailwinds are shown in normal font and headwinds are shown in bold font.

* Did not make stopover in Liberia.

## Discussion

Our data on swift migration provided the first complete migrations of this enigmatic species, which migration has been surrounded with so much myth and speculation [14], [15], [16], [19]. We can now answer several open questions regarding its migration route and strategy, which also have more general consequences for our understanding about migration strategies in birds in general, and in purely aerial bird species in particular.

### Migration Route and Wintering Area

The initial mean migration direction of 182° is identical to that of young swifts ringed in Sweden and recovered on autumn migration at least 10 km away from the site of ringing [19]. Hence, it seems as if adult and young swifts initially migrate in the same direction. Of the six swifts four shifted orientation in central or southern Europe to a SW direction towards the Iberian Peninsula, while the other two individuals that crossed the Mediterranean via the Balkan/Apennine Peninsulas also showed shifts towards a more westerly route when crossing the Sahara than that taken through Europe. All autumn migration routes involved a SSW migration direction to stopovers in West Africa, and none of the birds migrated directly to the final wintering area in central Africa (see more about detours below). Large numbers of swifts are observed during autumn migration in the river Niger inundation zone in Mali [21], an area where our swifts appear to pass.

All individuals stayed the winter in the Congo basin, with some minor shifts of winter location over the winter months. There are limited numbers of ringing recoveries reported from the winter period, with only one Swedish recovery from the Afrotropical region in Congo [19], while birds ringed in Britain/Ireland have generated recoveries as follows [18]: Congo basin (18), Malawi (11), Tanzania (2), Zambia (1), Zimbabwe (1) and Mozambique (1). A swift ringed in Switzerland has also been recovered in Congo during the winter [17], and three swifts ringed in the Netherlands have been recovered in Congo (1) and Tanzania (2) [22]. Taken together, we speculate that it seems as if Swedish swifts winter in the Congo basin, while British/Irish swifts appear to have a wider wintering area ranging from the Congo basin and towards southeastern Africa. However, available data are still limited and advice against definite conclusions, but the technique of geolocators holds great potential in describing possible population-specific segregation during the winter.

The spring route was similar to that of the autumn, but five of the swifts visited a restricted area (Liberia) in West Africa. The first leg of spring migration towards Liberia was more to the south than the corresponding leg during autumn migration, when the birds visited the Savannah zone [21]. The tracks support the hypothesis that terrestrial birds, such as the swift, may fly across the Gulf of Guinea in spring [23], [24]. One individual took a more direct route northwards across the Sahara, including crossing the Mediterranean at its widest part, with a direction change towards NNW after a stopover in the Aegean Sea area.

### Detours

The extensive detours of on average 53% and 43% during autumn and spring migration respectively, suggest there is some ecological advantage of migrating via West Africa instead of along a direct N-S axis. Detours can occur for several reasons, such as when the direct route involves the crossing of an ecological barrier (desert, sea or ice) where fuelling is not possible. A detour to avoid crossing the barrier, or one involving a shorter barrier crossing, may be favourable if the longer detour allows migration with smaller fuel reserves than needed for a direct flight across the barrier [25]. A detour may also be favourable if it allows faster fuelling at stopovers than for the direct flight, or if the transport cost is reduced due to for example tail winds [25]. Focusing on the spring migration, swifts experienced tail winds during both the initial migration leg to the stopover in Liberia, as well as during the subsequent Sahara crossing from West Africa. Generic wind patterns in the Sahara during spring are relatively stable [26], and thus swifts would predictably encounter tailwinds in the Western Sahara, whereas the central and eastern Sahara are dominated by headwinds. Furthermore, the timing of the stopover in Liberia coincides with the mass emergence of insects in connection with the onset of rains [24], which provides an opportunity for rapid fuel accumulation. Swifts have an exceptional capacity to forage and collect masses of aerial insects in a short time, and thus, potentially a capacity of fast increases in fuel reserves [26]. Hence, there are two ecological factors favouring the West African detour to allow for a fast spring migration. The swift taking a direct route from the wintering area towards N in spring experienced headwinds across the Sahara, resulting in a relatively slow progress, illustrating the possible cost associated with this more direct route.

The quadratic relationship between travel rate (including days of travel) and latitude indicates that relatively more time was devoted to directed flights, and less to en-route foraging, about latitudes 25–30°N. This pattern may also arise if wind assistance varies in relation to latitude. In fact, analysing travel rate in relation to latitude and wind assistance showed that both variables significantly contributed to the variation in travel rate, except for wind data at the lowest altitude (e.g. at 3000 m, Mixed model fixed effects; latitude F_1,106_ = 15.2, P = 0.0002, wind assistance F_1,105_ = 32.3, P<0.0001; latitude × wind assistance F_1,105_ = 11.7, P = 0.0009).

### Migration Strategy

The migration paradigm for passerines involves alternate cycles of stopovers for fuelling and flight [28]. Theoretical calculations suggest that small passerines should divide time on migration between flight and stopover at about 1∶7, which means that 87.5% of time is spent at stopovers [28]. A study using geolocators found that six passerines (two purple martins *Progne subis*, five wood thrushes *Hylocichla mustelina*) spent on average 64% of the time at stopovers, while the corresponding numbers for spring migration was 24% [11]. The swifts of this study spent on average 47% and 27% of the time at stopovers during autumn and spring migration, respectively. It should be noted that these numbers do not include the initial time for fuelling at the breeding/wintering site and that on days of travel less than 24 hours are likely spent flying in the migration direction, which therefore underestimates the time spent at stopovers. The pattern is similar between the seasons, reflecting a faster spring migration for both the passerines and the swift. Autumn migration was, however, faster for the swift and the purple martin (170 and 153 km/day, respectively) than for the wood thrush (68 km/day), suggesting the aerially feeding species achieve a substantially faster migration. However, the spring migration speed was on average 242 km/day in the wood thrush, which is very high for a passerine (cf. [30]), but still lower than in the swift and purple martin (336 and 429 km/day, respectively). The highest travel speed recorded for any of our swifts was 650 km/day, suggesting migration with wind assistance. Passerines, such as the wood thrush, probably accumulate large fat reserves at the winter site that allow such a fast spring migration (cf. [30]). Average summer body mass for swifts is about 40 g in southern England [26], depending on food availability as indicated by temperature. Body masses of our swifts at arrival in spring (42.5 g) and shortly before departure on autumn migration (43.2 g) did not suggest the accumulation of any extensive fuel reserves at these times. To the best of our knowledge there is no quantitative information about fuelling in the winter quarters before spring departure (cf. [31]). However, the fact that swifts do spend time at stopovers during migration suggests they exploit these areas for fuelling, especially since the stopovers appear to be located before the crossing of the Sahara and the Mediterranean (Fig. 1). This pattern was especially pronounced in spring, when five of the swifts stopped over in Liberia, involving a substantial detour. Why would they visit this restricted area in West Africa? The timing in late April and early May coincides with the onset of the rainy season and the associated emergence of aerial insects [24]. Gatter [24] writes that during this period “the skies can be full of Common swifts throughout the country on some days”, and out of more than 2 million swifts that he recorded during 1981–1994 only 7% were observed between August and December, while 92% were recorded from March to May. In April Gatter [24] also observed a “continuous movement” of swifts flying towards NW from an aircraft at an altitude of 1000 m above broken cloud in western Liberia, while at Mount Nimba departure directions were towards NNE during March-April. Even if swifts occur in significant numbers during April and May also in Ghana and Nigeria [32], [33], they do not appear to reach the numbers found in Liberia [24], which supports the notion that swifts to a large extent migrate across the Gulf of Guinea in spring as suggested by Gatter [24]. With such a concentration of swifts during spring migration to a relatively small area in West Africa, it follows that swift populations may be vulnerable to habitat loss there (cf. [34]).

A comparison with flight speed of swifts can inform about the migration strategy. The flight speed of swifts on spring migration in southern Sweden measured by tracking radar was 10.6 m/s [34], which corresponds to 916 km/day in the migratory direction if flying for 24 hours. The average travel rate was about half the flight speed in spring, suggesting that the swifts migrated for about 12 hours of the day and presumably foraged with slow or no progress for the remaining time. Highly aerial species that hunt food in the open air are predicted to adopt a fly-and-forage migration strategy or to combine fly-and-forage with stopovers (mixed strategy) [8]. The fly-and-forage strategy will be favoured if
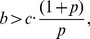
where *b* is the relative benefit from en-route foraging as reduced effective flight power consumption, *c* is the cost as reduced travel speed due to foraging, and *p* is the power ratio (*P*_trav_/*P*_dep_, where *P*_trav_ is power required during travelling (flight) and *P*_dep_ is rate of energy accumulation at stopover [8]. Hence, a fly-and-forage strategy is favoured if *b* is relatively large, *c* is small or *p* is large, or a combination of these factors satisfying the inequality. A high *b* and low *c* are likely satisfied by the swift, since it can forage in flight during migration with a small reduction in travel speed, while the power ratio is likely to be low. Swifts have an efficient aerodynamic design that will give a relatively low power required to fly [35]–[37], so a high power ratio will depend on the energy deposition rate. In some circumstances swifts can gain weight very quickly [27], but this rate depends on food availability (temperature) and the effort needed to search for food and may therefore vary a great deal. Taken together, the swift possesses features that would make a (mixed) fly-and-forage strategy beneficial to reach an overall migration speed that is much higher than a stop-and-fly strategy. Notice that stopovers are part of a mixed fly-and-forage strategy, i.e. the fact that a bird makes a stopover does not mean that it uses a fly-and-forage strategy for the rest of its migration. Especially before the crossing of wide ecological barriers birds using a fly-and-forage strategy might make stopovers, which seems to be the case in the swift before the Sahara on both autumn and spring migration.

Because the swift can combine foraging and migration and since it can sample food abundance continually during migration, it will probably not experience the search/settling time and energy costs of avian migrants that depend on terrestrial stopovers [20], [29], [38]. Being adapted to a life in airspace, the swift has a low-cost aerodynamic design and a comparatively high effective lift to drag ratio [36], [37], [39] that minimize the cost of transport. These factors in combination constitute the features that allow the swift to migrate so exceedingly fast, when compared to other less aerial species (cf. [30]). Interestingly, the purple martin, which is also a species of efficient aerodynamic design, also exhibits a relatively high migration speed [11].

### Annual Cycle

Based on our migration tracks of swifts the average annual time allocation could be estimated for breeding, migration and wintering as 19%, 27% and 54%, respectively. Autumn migration took longer time than spring migration, which may be explained by differing strategy between the seasons (see above). More than half the year is spent on the wintering grounds, presumably without coming to the ground except during rare occasions [15]. Also our light transition data indicate that the swifts are airborne throughout the northern winter as we never observed any false twilight events caused by shading by feathers or vegetation, which are typical for geolocator data from species in more closed or forest habitats [40]. Interestingly, the duration of the wintering period equals the duration of wing feather moult, which also takes about six months [41].

### Conclusion

Using gelocators we have been able to reveal the details of the migrations of six Swedish swifts. Despite a small sample size, we have learned more about migration routes, wintering areas, timing of migration, travel rates and migration strategies of this species than from a century of bird ringing. For example, not a single ring recovery is known from any European ringing schemes from the Liberia region, which appears to be a major stopover area in spring. Taken together, the swift has many analogies with seabirds. Just as seabirds, swifts live in an environment where food may be found virtually everywhere. Furthermore, swifts have a mixed fly-and-forage strategy, making stopovers in areas where feeding conditions are likely to be outstanding, such as Liberia in spring. This is very similar to seabirds that probably combine migration and foraging to a large extent, but also make stopovers in areas with high food abundance [9], [10].

## Materials and Methods

We attached eight archival Mk10 geolocators from the British Antarctic Survey (BAS) to common swifts in two breeding colonies in southern (N = 6; 55.47°N, 13.50°E) and central Sweden (N = 2; 60.28°N, 18.26°E) in May, July and August 2009. In 2010 six of the geolocators were recovered (5 in South Sweden and 1 in Central Sweden). This is comparable to published data on annual survival rates in common swifts at about 80% [42], [43]. We attached the geolocator with a full body harness, consisting of two nylon strings from the geolocator (positioned dorsally between the wings) forming a loop around the neck, where a knot on the ventral side fixed them. From this knot each of the two strings go backward under and around the wing and back to the geolocator where they are fixed to the geolocator on the back of the bird. Hence, the harness forms three loops, one around the neck and one around each wing. One of the swifts was used in a pilot trial where the geolocator was attached shortly after the arrival from spring migration on 20 May 2009, and we monitored the breeding of this bird throughout the summer using a camera in its nest. The bird fed the young at a normal rate and breeding was successful resulting in 2 chicks surviving until fledging. Hence, the geolocator did not seem to negatively affect the bird during the breeding season. The remaining 7 geolocators were attached to adult swifts at the end of the breeding period shortly before the young left the nest. The birds were captured in the nest boxes (southern Sweden), or with a mist net outside the nesting site in central Sweden. At capture and recapture we recorded the weight of the birds (to the nearest 0.1 g) with a spring balance, except for one bird where we failed to measure the mass at first capture. The mean body mass at capture was (mean ± SD) 43.2 (±2.3) g and at recapture it was 42.5 (±2.8) g, but the difference was not significant (Matched pair test, t = 0.90, P = 0.4, N = 5). The mass of the geolocator was 1.3 g including harness and glue, which amounts to 3% of the body mass of the bird. When handling the birds on recapture we could not detect any signs of feather or skin abrasion due to the geolocator harness, or any other negative signs from carrying the geolocator. Birds trapped but not used in this study were ringed and released. Permissions to trap swifts at the study locations were obtained from the landowners.

Light-level data were linearly corrected for clock drift using the program BASTrak [44], and times of sunrise and sunset were extracted using the program TransEdit [44] using a single light threshold value of 2. Positions were calculated with the BirdTracker software [44], in which latitude was inferred from the length of the solar day/night and longitude from the time of local solar noon/midnight, respectively. For these calculations we used a critical sun angle (i.e. the sun angle corresponding to a light-level value of 2 on the arbitrary BAS geolocator light scale) that minimised the difference in latitude between pre- and post equinox, and simultaneously the uncertainty in latitude close to equinox for periods when the birds were stationary (as deduced from longitude). This ‘Hill-Ekstrom’ procedure is based on the observation that around the equinoxes the error in latitude increases with increasing mismatch between light threshold value and inferred sun angle [45]. The appropriate sun angle can be determined by calculating latitudes for a range of candidate sun angles and selecting the one that minimizes the variation before and after the equinox [45]. For a comprehensive explanation and evaluation of this and alternative calibration methods see ref. [46]. Sun angles used varied from −6 to −7 degrees. Data on latitude were excluded for approximately 14 days before and after vernal and autumnal equinox.

Overall, we obtained two positions per day, and both midnight and noon locations were used in our analyses. We distinguished between movements and stationary periods (migratory stopovers, breeding, wintering) by inspecting subsequent positions. Due to the inaccuracy of positioning data, stopovers shorter than two days could not be distinguished from slow movements. For further analysis and plotting we calculated 3-day mean positions (i.e. means for 6 subsequent position estimates). Total migration distance is the sum of the length of segments based on 3-day means, in which stationary periods are excluded. The direct distance is the Great Circle Route between breeding and wintering site.

The period over which to calculate mean positions affect the estimated migration distance and derived properties such as detour and migration speed. We consider 3-day means for the positional data as a reasonable compromise between using shorter periods that will inflate migration distances due to noise in the data, and using longer periods that will underestimate the true migration distance due to the omission of real movements away from the straight line between successive positions. To illustrate the effect of period on the estimated migration distances we calculated mean positions for 1-day, 2-day and 5-day periods in addition to the 3-day means used for the analyses for autumn and spring migration, respectively. As expected, 1-day and 2-day means resulted in increased estimated migration distance compared with 3-day means by 37% and 8.6%, respectively, while 5-day means resulted in reduced migration distance by 5.4% compared with 3-day means (percentages are means for all the six swifts). The corresponding numbers for estimated migration distances for spring were increase by 36% and 15% (1-day and 2-day means), and a decrease by 7.8% (5-day means), when compared with 3-day means. The effects on derived properties were very similar.

Positions derived from light-level geolocators are marred with errors of magnitudes estimated at 143±62 km (mean ±95% confidence interval) and 186±114 km (mean ± SD) for latitude position [40], [47], respectively, depending on factors such as geographical region, time of year, habitat and weather. Errors of longitude estimates are generally lower than those of latitude, estimated at 50±34 (mean ±95%confidence interval) and 85±47 km (mean ± SD) [40], [47]. Errors in positional estimates may affect derived properties such as migration and travel rates. Most errors in geolocation by light are caused by shading events; i.e. due to the shading by feathers, vegetation, and clouds. The sun seems to rise later or sets earlier than expected. Shading events affect estimates of the length of solar day (and night) and time of local solar noon (and midnight), and consequently result in errors in positional estimates. When determining longitude, there will be no error from shading if the same amount of shading is present at rise and set. If the amount of shading differs, the derived local noon/midnight will be shifted from true and there will be an associated error in derived longitude. For latitude, where day/night length is the input, if the shading experienced differs from that which formed the relationship between light level threshold and sun altitude through calibration, there will be an error in latitude. In addition, any movement of the bird between rise/set and set/rise will alter the derived day/night length and local noon/midnight in a manner that is unlikely to result in the mean position for the bird. The dominant error due to movement is with calculated latitude but is cancelled if consecutively derived noon and midnight latitudes are averaged [44], as we have done. In the present analysis (following [48]), distance estimates are based on 3-day averages, i.e. 6 subsequent positional estimates for each location, which reduces the errors in location estimates. Furthermore, the plotted migration routes (Figure 1) show relatively straight movement segments, which suggest that estimated travel distances and rates have not been inflated due to precision errors. Notice that errors arising from an incorrect calibration of threshold light level with sun altitude are largely systematic and so have far less effect on speed estimates than on actual location.

For the wind analysis, location data were smoothed twice (cf. [49]), giving weighted estimates for noon and midnight positions, which were used to define 12 h-segments. Wind data (speed and direction of the wind) were obtained from the NCEP/NCAR Reanalysis project, as provided by NOAA/OAR/ESRL PSD, Boulder, Colorado, USA (http://www.cdc.noaa.gov). For every 12 h-segment, wind data were extracted for the start, mid- and endpoint, in which the midpoint was given twice as much weight during averaging. We subsequently calculated, for every segment, the tailwind component of the average wind vector, which is the amount of wind blowing parallel to the general migration direction (i.e. the projection of the wind vector on the general axis of migration). For the spring migration, two general migration directions were defined, the direction from the wintering area to the stopover area in Liberia, and the direction from Liberia to the breeding site. For individual ‘7964’, which did not make a detour via Liberia, a single general migration direction was used (direction from wintering area to breeding site). The amount of tailwind experienced by the swifts was averaged per individual and per travel leg (wintering area – Liberia, crossing of the Sahel and Sahara Desert, crossing of Europe). Negative tailwind values represent headwinds. These calculations were repeated for different pressure levels (925, 850, 750 and 500 hPa), which correspond to different altitudes (750, 1500, 3000 and 5000 m, respectively).

## Supporting Information

Figure S1 **Maps showing migration routes, breeding sites and wintering areas for the individual common swifts tracked using light-level geolocators.**
(PDF)

Table S1 **Key numbers of migration and wintering for individual common swifts tracked by light-level geolocators.**
(DOCX)
