# Supplementary material for: Migration Routes and Strategies in a Highly Aerial Migrant, the Common Swift Apus apus, Revealed by Light-Level Geolocators
Source: PLoS One. 2012 Jul 18;7(7):e41195. doi: 10.1371/journal.pone.0041195 (PMC3399846; doi:10.1371/journal.pone.0041195)

Figure S1. Maps showing migration routes, breeding sites and wintering areas for the individual common swifts tracked using light-level geolocators. Red lines show autumn migration tracks with filled circles representing 3-day average position, and with larger yellow circles indicating sites of stopovers during at least 2.5 days (estimated stopover duration is indicated by numbers). Winter locations are shown with blue circles, and for three individuals that shifted locations during the winter period the alternative wintering area are shown by triangles. Wintering areas were as follows: 7881 (1 wintering area): 10 September- 26 April; 7882 (2 wintering ares): 19 November- 8 January, 9 January- 30 April; 7964 (2 wintering area): 31 October – 7 December, 10 December- 23 April; 7968 (1 wintering area): 27 August- 27 April; 7969 (3 areas, area 1 = area 3): 10 October- 11 December, 16 December- 23 January, 8 February- 27 April; 7970 (1 area): 22 October – 24 April. Green lines show spring migration tracks, but otherwise same as for autumn migration.

7881

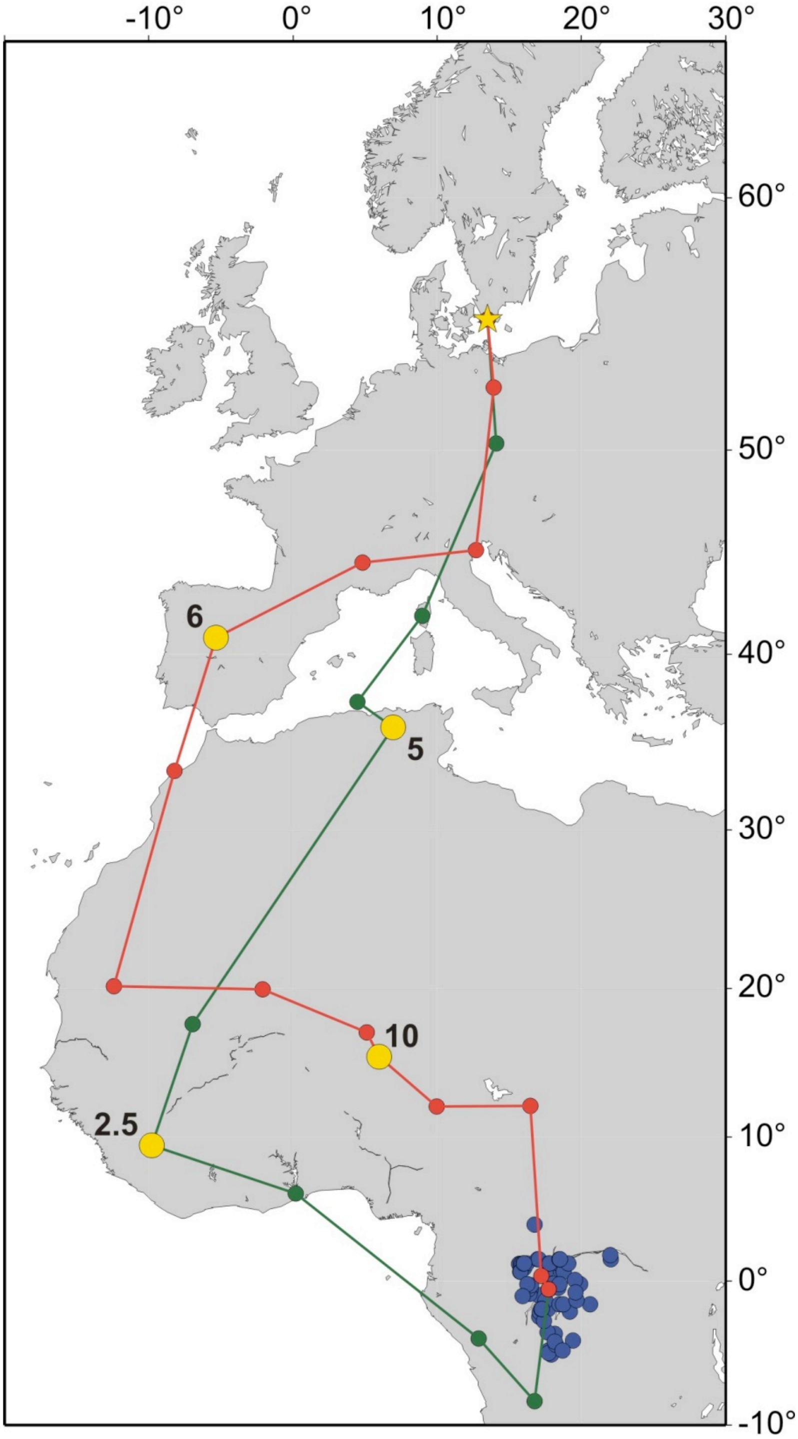

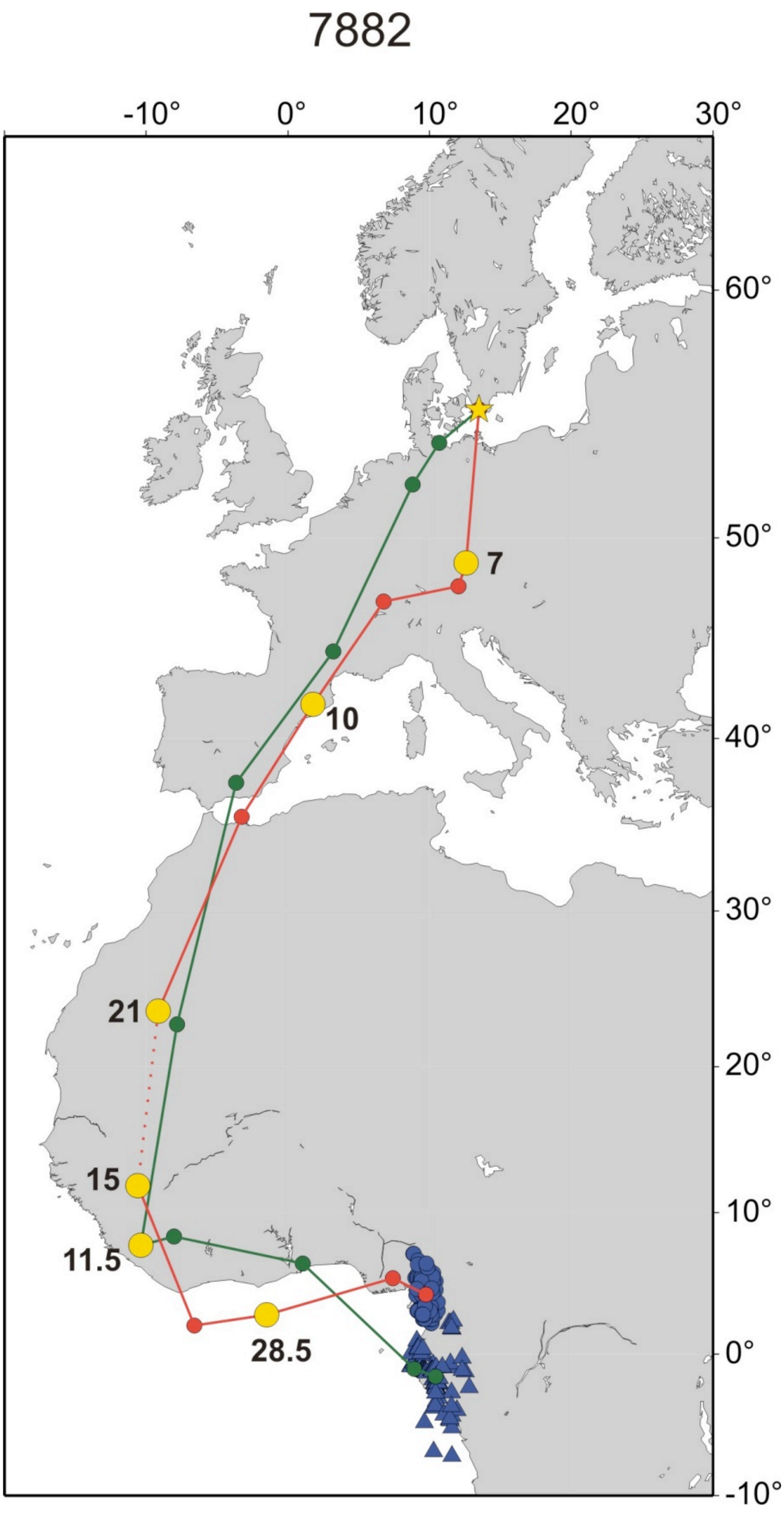
7882
-10°
0°
10°
20°
30°
60°
50°
40°
30°
20°
10°
0°
-10°
7
10
21
15
11.5
28.5

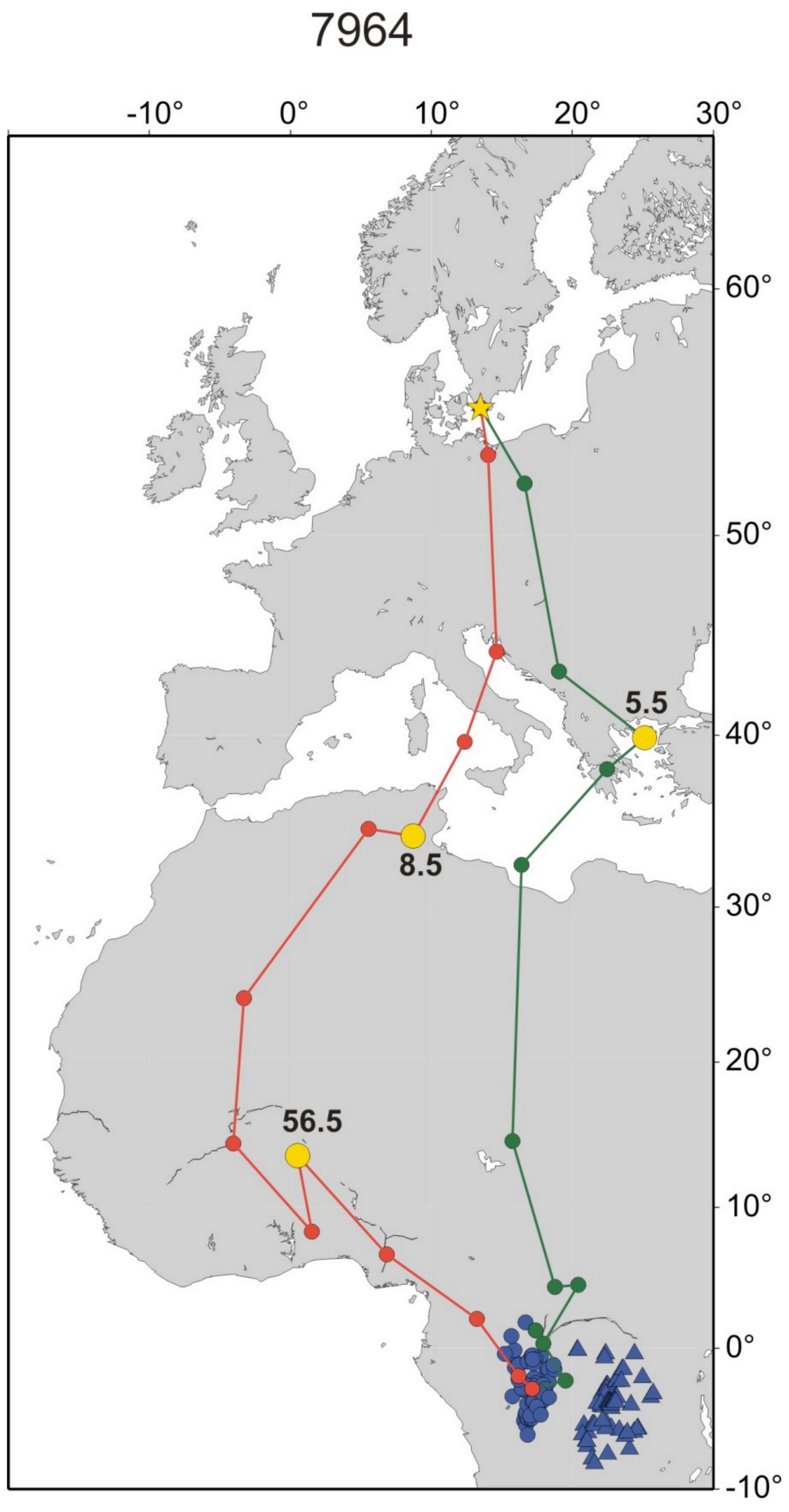
7964
-10°
0°
10°
20°
30°
60°
50°
40°
30°
20°
10°
0°
-10°
5.5
8.5
56.5

7968

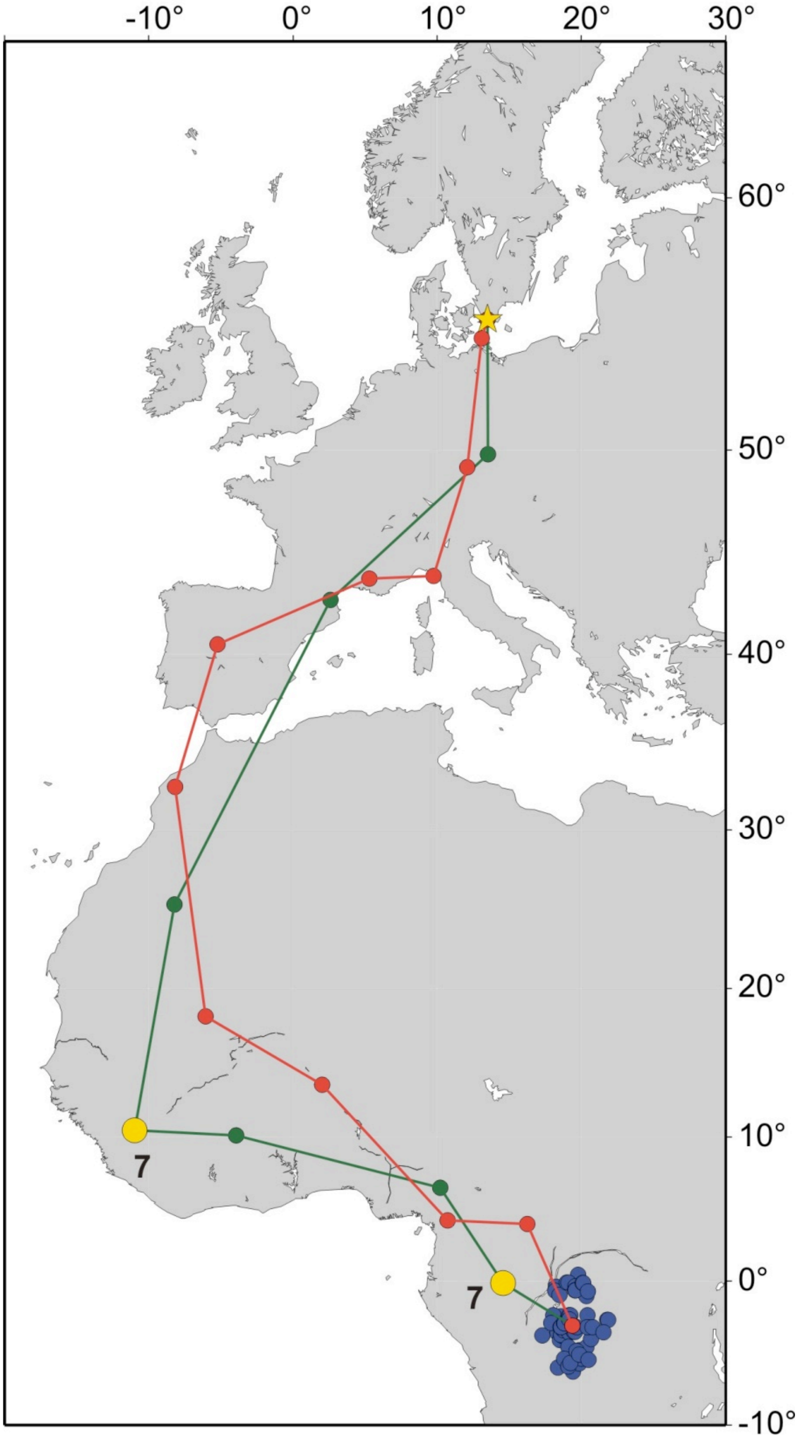

7969

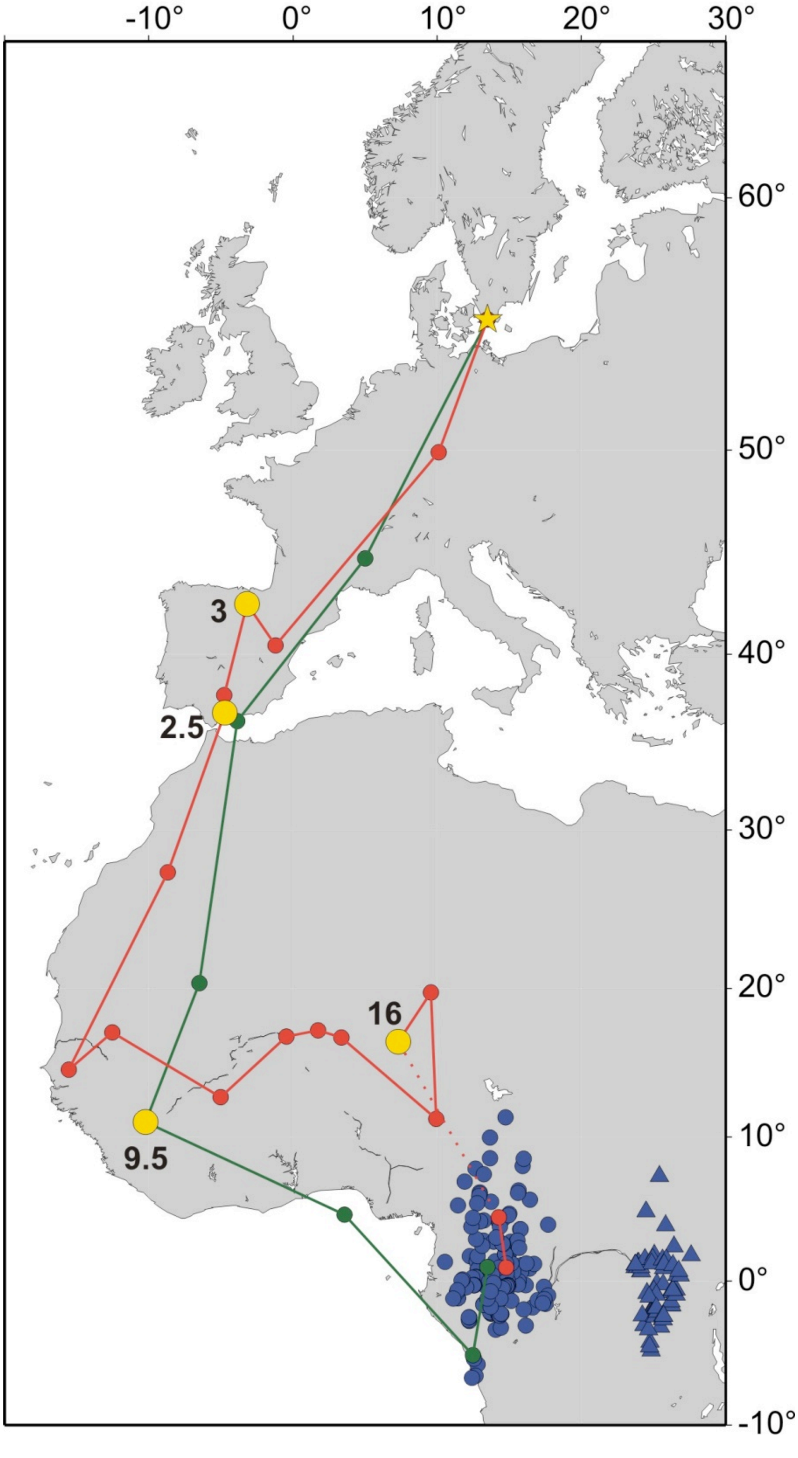

7970

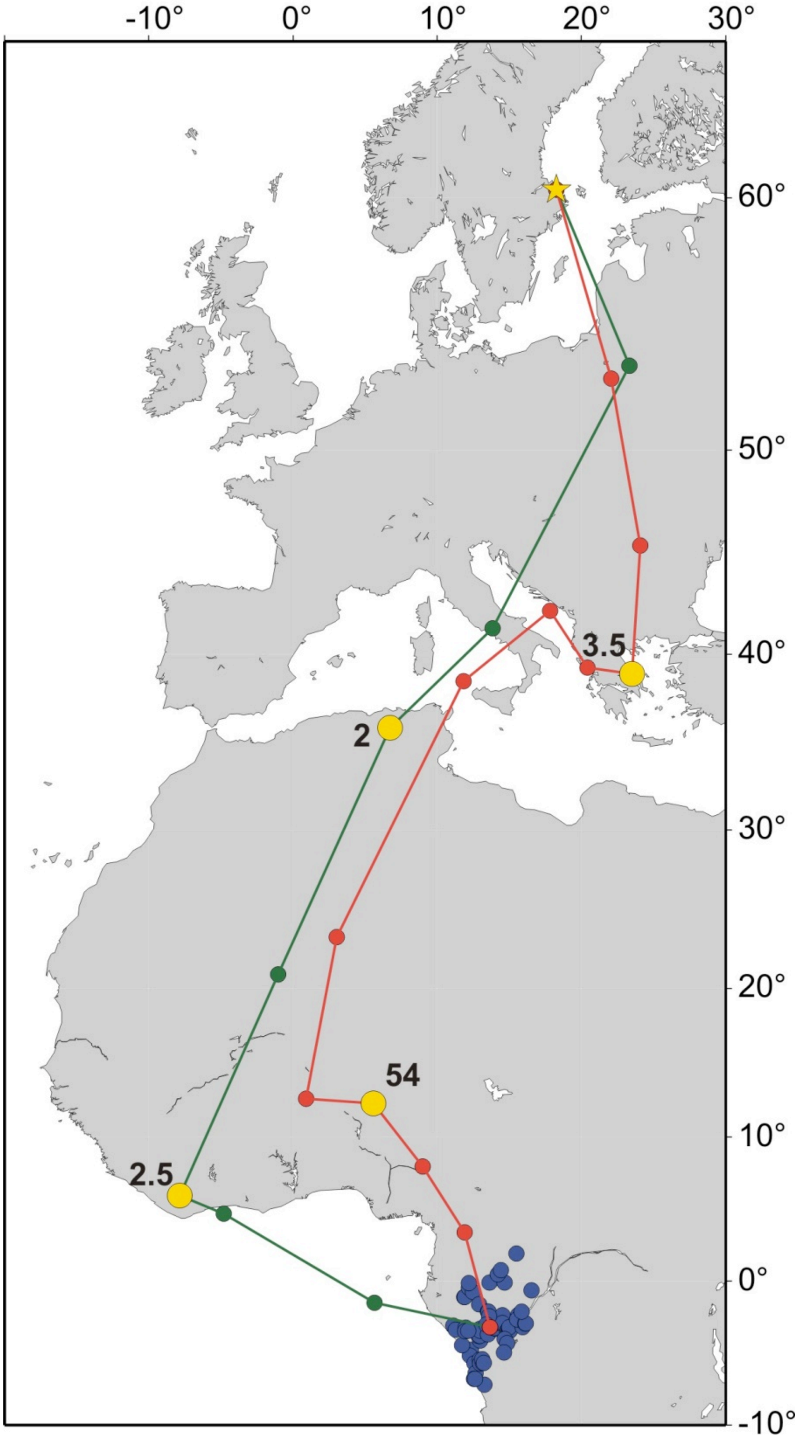

Supplement: Figure S1 — Maps showing migration routes, breeding sites and wintering areas for the individual common swifts tracked using light-level geolocators. (PDF) [file pone.0041195.s001.pdf]
