# Supplementary material for: Migration Routes and Strategies in a Highly Aerial Migrant, the Common Swift Apus apus, Revealed by Light-Level Geolocators
Source: PLoS One. 2012 Jul 18;7(7):e41195. doi: 10.1371/journal.pone.0041195 (PMC3399846; doi:10.1371/journal.pone.0041195)
Supplement: Table S1 — Key numbers of migration and wintering for individual common swifts tracked by light-level geolocators. (DOCX) [file pone.0041195.s002.docx]

| **Autumn migration** | **7881** | **7882** | **7964** | **7968** | **7969** | **7970** | **Average** |
| --- | --- | --- | --- | --- | --- | --- | --- |
| Departure from breeding area (date) | 30 July | 12 Aug | 31 July | 28 July | 4 Aug | 29 July | 2 Aug |
| Travel time (days) | 27 | 18 | 28 | 30 | 47 | 29 | 30 |
| Stopover time (days) | 15 | 82 | 64 | 0 | 20 | 56 | 39 |
| Total duration (days) | 42 | 99 | 92 | 30 | 67 | 86 | 69 |
| Arrival at wintering area (date) | 10 Sep | 19 Nov | 31 Oct | 27 Aug | 10 Oct | 22 Oct | 10 Oct |
| Total migration distance (km) | 9900 | 8629 | 9438 | 9031 | 12380 | 9238 | 9769 |
| Direct distance (km) | 6247 | 6352 | 6499 | 6535 | 6061 | 6937 | 6439 |
| Difference Total-Direct (%) | 58% | 36% | 45% | 38% | 104% | 33% | 53% |
| Travel rate (km/day) | 367 | 481 | 331 | 302 | 263 | 318 | 344 |
| Migration rate (km/day) | 236 | 87 | 102 | 302 | 185 | 108 | 170 |
|  |  |  |  |  |  |  |  |
| **Wintering period** |  |  |  |  |  |  |  |
| Duration of wintering period (days) | 228 | 162 | 174 | 243 | 199 | 184 | 198 |
|  |  |  |  |  |  |  |  |
| **Spring migration** |  |  |  |  |  |  |  |
| Departure from wintering area (date) | 26 Apr | 30 Apr | 23 Apr | 27 Apr | 27 Apr | 24 Apr | 26 Apr |
| Travel time (days) | 23 | 23 | 29 | 17 | 19 | 14 | 21 |
| Stopover time (days) | 6 | 11 | 5 | 13 | 9 | 4 | 8 |
| Total duration (days) | 29 | 34 | 34 | 30 | 29 | 18 | 29 |
| Arrival at breeding area (date) | 26 May | 2 June | 27 May | 26 May | 25 May | 12 May | 25 May |
| Total migration distance (km) | 10390 | 8457 | 7946 | 9524 | 9515 | 9416 | 9208 |
| Direct distance (km) | 6247 | 6352 | 6499 | 6535 | 6061 | 6937 | 6439 |
| Difference Total-Direct (%) | 66% | 33% | 22% | 46% | 57% | 36% | 43% |
| Travel rate (km/day) | 452 | 376 | 274 | 577 | 488 | 650 | 469 |
| Migration rate (km/day) | 352 | 252 | 234 | 323 | 334 | 523 | 336 |

Table S1. Key numbers of migration and wintering for individual common swifts tracked by light-level geolocators
